# Training the eye, virtually: adapting an art in medicine curriculum for on-line learning

**DOI:** 10.1007/s43545-022-00442-4

**Published:** 2022-08-11

**Authors:** Ambike Aarti Srivastava, Stephanie Cohen, Dabney Hailey, Shahram Khoshbin, Joel T. Katz, Ingrid M. Ganske

**Affiliations:** 1 Harvard Medical School, Boston, MA USA; 2 Department of Surgery, Beth Israel Deaconess Medical Center and Harvard Medical School, Boston, MA USA; 3 Harvard Medical School and the Hailey Group, Boston, MA USA; 4 Department of Neurology, Brigham and Women’s Hospital and Harvard Medical School, Boston, MA USA; 5 Department of Internal Medicine, Brigham and Women’s Hospital and Harvard Medical School, Boston, MA USA; 6 Department of Plastic and Oral Surgery, Boston Children’s Hospital and Harvard Medical School, 300 Longwood Avenue, Enders 123, Boston, MA 02115 USA

**Keywords:** Visual thinking strategies, Art in medicine, Virtual education, Medical education

## Abstract

Training the Eye: Improving the Art of Physical Diagnosis is an elective fine art-based medical humanities course at Harvard Medical School held at the Museum of Fine Arts, Boston that aims to improve skills of observation. Due to COVID-19, this curriculum was converted from in-person to a virtual format for the first time in 2020. Students enrolled in the course prior to the pandemic and completed one session in person before transitioning unexpectedly to nine remote sessions through Zoom. Students were surveyed anonymously and TAs and faculty were interviewed regarding their perceptions of the strengths, weaknesses and future preferences of the virtual arts education at the course completion. Strengths identified in the virtual platform were being able to include participants irrespective of their location, incorporating most relevant artwork from any collection, harnessing virtual tools for enhanced art viewing, time-efficiency, and having a private, safe space for engaging in this type of learning. However, the experience in the galleries and the social interactions therein were noted to be impossible to fully recapitulate. Personal connections of the class were felt to be diminished and convenience increased. Both advantages (e.g., increased reach relative to types and locations of art works) and disadvantages (e.g., intimacy and connectivity promoted by in-gallery setting) of teaching arts-based medical humanities virtually were identified. A hybrid model may be able reap the benefits of both formats when it is safe to host such courses in person. Parallel lessons may be applicable to optimizing telemedicine encounters.

**Supplementary Information:**

The online version contains supplementary material available at 10.1007/s43545-022-00442-4.

## Introduction

In the Spring of 2020, medical schools around the world were required to rapidly restructure curricula to be suitable for virtual teaching platforms during the COVID-19 global pandemic. For 18 years, an art museum-based physical examination course, “Training the Eye: Improving the Art of Physical Diagnosis” (TTE) has been taught at Harvard Medical School (Naghshineh et al. 2008). The purpose of this paper is to: (1) describe the modifications that were made to this course; (2) summarize the narrative experiences of the students, teaching assistants (TAs) and faculty; (3) report the post-course assessment provided by class participants; and 4) discuss lessons learned that could improve and optimize virtual teaching for similar courses.

TTE is a 10-session, weekly physical examination (PE) elective course primarily for medical and dental students that is taught at a local art museum; a limited number of undergraduate and graduate students can enroll if space permits. The course teaches visual observation skills—the most fundamental aspect of the PE—through the structured analysis of art to aid in the development of unbiased diagnostic and communication skills that can be directly applied to patient care. The primary course objectives are for students to develop a routine practice of careful and objective looking, gain confidence in connecting form with function and effectively communicating these observations. The secondary objectives include promoting mindfulness, community building, and improving cultural dexterity and perspective-taking through exposure to a wide breadth of artwork.

TTE uses a teaching methodology called Visual Thinking Strategies (VTS), which was co-developed by cognitive psychologist Abigail Housen and museum educator Philip Yenawine (Housen 1992); Housen 2002). In VTS, participants openly express their own observations and subjective interpretations of a particular work of art. Group members actively build on each other’s viewpoints and dynamically reshape their interpretations. VTS has been used to teach visual literacy, critical thinking, and communication skills, all of which are relevant to the field of medicine (Reilly et al. 2005). Studies have demonstrated that learners from different domains and specialties benefit from VTS and variations on this type of art-based pedagogy (Dolev et al. 2001; Jasani and Saks 2013; Klugman et al. 2011; Shapiro et al. 2006; Shapiro and Shallit 2014).

The TTE curriculum is an important part of emerging medical humanities curricula (Belling 2010; Krasner et al. 2009; Wear and Zarconi 2006). Art, poetry, narrative essays, music, and theater encourage and motivate compassion and empathy in medicine (Brainard and Brislen 2007; Schaff et al. 2011; Shapiro 2002). Unfortunately, during the pandemic, medical humanities courses have faced unique hurdles in adapting to remote learning platforms. We reviewed the 2020 TTE course to understand the impact of how the course was adapted, assess the effect of these changes on attainment of the course goals, and catalogue lessons learned.

## Methods

### Course adaptation

The first session of the TTE course met in person on March 6, 2020, just prior to the Association of American Medical Colleges (AAMC) recommendation for cessation of on-site academic activities and group gatherings. This session followed the traditional model of introductions of the faculty and students with overview of the course in the medical school classroom, followed by a short walk to the Museum of Fine Arts, Boston (MFA), where a faculty member (DH) introduced the VTS framework and led the group through an observation exercise. By the following week, nearly all medical school courses were converted to virtual formats. The syllabus was recapitulated nearly directly for its new format on Zoom (Zoom Video Communications Inc., San Jose, CA) (Table 1). Whereas each session had typically included an on-site lecture and art observation session at one of the local museums (Museum of Fine Arts, Boston; Isabella Stewart Gardener Museum; Harvard Art Museum), instead the lectures were given virtually and VTS sessions were done in small Zoom break-out rooms, using pieces of art from collections around the world, rather than just the art that is locally on view. Student-led VTS sessions were a core component of the course pre-COVID and continued to be integral in the on-line version of the course as well. A pass/fail grade was based on student attendance, engagement, and completion of sketchbook and journal responses, just as it had been in prior years.

**Table 1:** Adaptation of 2020 TTE course

Course component	Traditional course plan	Adapted course plan
*Lecture/VTS Lesson (7 sessions)*		
Lecture	Given at Medical School or Museum, in small lecture hall	Given “live” on Zoom, student participation encouraged
Transit	Walk to museum	Not applicable
Gallery drawing	In galleries, including both independent and collaborative exercises. Homework also included drawing exercises	Drawing component of weekly schedule limited to independent homework assignments
VTS sessions	In small groups (4–7 students) in the museum galleries, with one TA and one faculty member per group	In small groups (4–7 students) in Zoom break out rooms, with one TA and one faculty member per group
Session wrap-up	Group reconvenes together to share insights and clinical correlations from the small group sessions. Updates from TA’s on weekly assignments	Group reconvenes in large Zoom meeting to share insights and clinical correlations from the small group sessions. Updates from TA’s on weekly assignments
*Lecture/Patient Visit Lesson (3 sessions)*		
Lecture	Given at medical school or museum, in small lecture hall	Given “live” on Zoom, student participation encouraged
Patient(s) Visit	A patient or panel of patients attend the lecture hall for a facilitated examination and observation exercise on clinical findings	Two patient panels were cancelled, due to condition of patients in COVID. The other was held virtually on Zoom, in a “telehealth” type of history and examination
*Life Drawing Lesson (1 session)*	In-person session with instructor and live model at Mass College of Art. Students perambulate to see each other’s work	Instructor “live” on Zoom, using pre-recorded drawing examples and pre-recorded model poses. Student participation encouraged; limited ability to share work
*Physical Diagnosis Rounds (2 sessions, separate from regular course lessons)*	Small group ward rounds with one of the course directors to practice observation skills in a clinical setting	Unable to incorporate this in 2020

Key differences in the course included the basic experience of on-line learning compared to in-person, with limited opportunities for the casual social connections and enriching conversations that take place in walking through the galleries together; the different pace of transitional time between activities in on-line sessions; and the distractions that may have simultaneously been present on-line for students.

Since the course was unable to view art pieces in the museum in person, we used high-resolution images of artwork selected from museums around the world. Students led VTS exercises while screen sharing the piece being discussed. While in-person VTS sessions include having a group facilitator standing at the front of the artwork and using body language and gestures to help paraphrase and clarify the group comments, on Zoom, instead, the facilitator had the ability to zoom in and out on the image and use the cursor throughout the discussions.

Some components of the course had to be cancelled. In typical years, students met in small groups with one of the course faculty to make rounds in the hospital and practice VTS skills on patients; however, when this course was conducted in the early phase of the pandemic, this activity was not possible. There are also usually at least three sessions of the course in which patients visit the whole class (e.g., gait abnormalities, breathing abnormalities, final class wrap-up). Attempts to shift these to the Zoom platform were unsuccessful due to a combination of patient limitations and technology issues; only one patient was able to participate in a remote visit that simulated a telehealth visit, moderated by one of the course directors.

### Course evaluation

To understand and improve the TTE course, students, TAs and course directors were surveyed about the modified format. Students were administered the standard, annual anonymous post-course questionnaire which they had time to complete during the last class (Appendix A). This was supplemented with additional open ended and 5-point Likert scale survey questions specifically about the virtual nature of the class which were distributed via anonymous Survey Monkey after course completion (Appendix B).

Four TAs per year are invited from among interested course graduates; they receive advanced VTS training to co-teach the small group sessions and receive a small financial stipend. One of the course TAs (AAS) conducted interviews with each faculty member and the three other TAs to gather perspectives of these participants. There are four faculty course directors, three of whom are physicians and one is a professional arts educator. Questions for the teaching assistants and faculty included: ease and effectiveness of the virtual format, technical challenges, impact on attaining the course objectives, perceived advantages with the on-line offering, suggestions for course improvement, and preference for fully or partially on-line format in future years. (Appendix C) Written notes in response to each question were recorded during the interviews.

Responses to open-ended questions on students’ written questionnaire’s and to the open-ended questions in TA and faculty interviews were reviewed and thematically grouped by the interviewer (AAS). Each course faculty member reviewed all questionnaire and survey comments as part of quality improvement for the course, as well as to confirm the thematic groupings. Student responses to Likert-scale questions in the post course survey were tallied and averaged.

## Results

### Students

The course participants included 29 students (10 male, 19 female); 4 TAs (1 male, 3 female), and 4 faculty (2 male, 2 female).

Comments on the post-course evaluation were similar to previous years, expressing three common themes: (1) An appreciation for deep looking (*“I will critically question why I am thinking a certain way and what more I can find.” “The questions of ‘what makes me say that’ and ‘what more can I see’ will absolutely help me slow down and notice things about patients that I might have otherwise missed.*) (2) A greater understanding of visual biases (“I will also always remember the VTS method and questioning why I see what I see.” *“I will try to put my findings in buckets of things that support my diagnosis and those that do not.”*) (3) Appreciation for the mindfulness aspects of the humanities curriculum *(“This was such a breath of fresh air from regular medical school courses and yet I learned so much about medicine from it.” “Especially given the limitations of the pandemic, the opportunity to see new works of art and discuss them with peers was greatly welcome.”*).

Of the 19 participants, 12 (63%) completed the additional survey regarding the on-line learning environment. (Table 2) Qualitatively, students reported that there were some advantages of using Zoom, such as using the cursor as a pointer to direct everyone’s attention to a particular part of a painting and being able to analyze art from around the world. However, they desired more interactive, socially connected sessions and shortened didactics, reporting that it was additionally hard to remain attentive for the full 2.5 h sessions. Sixty percent of students agreed or strongly agreed that the TTE curriculum worked well over Zoom, 30% were neutral, and 10% somewhat disagreed. Seventy-five percent of students agreed or strongly agreed that the course objectives were able to be met virtually, 8 percent were neutral, and 17 percent disagreed.

**Table 2:** Advantages and disadvantages of adapting the course to the Zoom platform

Cohort	Advantages and new opportunities	Disadvantages and challenges
*Students*	***Technical:***	***Technical:***
	•Features of Zoom aided visualization of the art (magnification, using cursor as pointer)	•Certain observations were difficult (scale, lighting, emotion, texture, dimension, etc.)
	***Logistical:***	***Logistical:***
	•Increased accessibility and convenience (no travel time or distance issues) •Minimal transition time meant more opportunities to lead and facilitate VTS	•Difficult to be present and concentrate in home environment versus immersive museum setting
	***Experiential:***	***Experiential:***
	•Welcomed opportunity for connection and discussion during an isolating pandemic	•Zoom lectures too long (fatigue) •Inability to personally engage with other students and faculty
*Teaching Assistants*	***Technical:***	***Technical:***
	•Features of Zoom aided visualization of the art (magnification, using cursor as pointer) •Everyone had the same, unobstructed view of the subject through screenshare	•Difficult to assess student engagement and presence virtually
	***Logistical:***	***Logistical:***
	•Wide selection of art available •No background noise as in galleries •No issues with planned art not being available for group use •We could make observations in our own space, less fear of judgment	•It was difficult to give subtle cues or reminders to students to help them in leading VTS sessions and required more direct guidance
	***Experiential:***	***Experiential:***
	•Ability to continue the course, despite mandatory social distancing •Being in one’s home environment created safe space to practice VTS, rather than with audience in the galleries •Student skills seemed to progress similarly as in the traditional course of years past	•Difficult to engage with students in TA mentorship role in virtual format •Lack of in-person interaction was a missed opportunity for meaningful personal connections and shared ideas •Less serendipity and important tangential experiences
*Course Directors*	***Technical:***	***Technical:***
	•The patient visit through the Zoom platform gave students some insight into telehealth experience and technology	•Difficult to assess body language and student reactions virtually •Certain observations of the art were more difficult (dimensionality, colors, scale, texture)
	***Logistical:***	***Logistical:***
	•Accessibility, especially to students from afar and busy frontline workers, including the faculty and lecturers •Expanded access to different media types and collections from all over the world	•It was more difficult to integrate the clinical experiences in the on-line format (physical diagnosis rounds, Patient Visit sessions)
	***Experiential:***	***Experiential:***
	•On-line platform helped break down hierarchies •Student skills and journal reflections seemed to progress similarly as in the traditional course of previous years	•Difficult to recreate the casual conversations and experience of walking through the galleries on-line

### Teaching assistants

The TTE TAs play a critical role in helping oversee the student experience, and they also have the additional goal of practicing their own teaching and facilitation skills. Thus, the TA perspective is two-fold: how well did the virtual course work in meeting student objectives, and how well did the course support the development of their skills in medical education. The four TAs noted advantages and disadvantages of the virtual version of the course in both regards. (Table 2) They also had additional insight into the alternative version of the course, having all taken the course in person in previous years.

The TAs noted logistical advantages for the student experience on-line, including a lack of distracting background noise in the galleries, unobstructed views of the artwork, and reliability that the artwork planned for the session would be available (in the galleries, preselected artwork occasionally had been moved or was occupied by another group, requiring live adaptation of course plans). They also noted that the remote setting was more private for our group, which fostered an environment in which students seemed to feel more comfortable expressing their thoughts without fear of judgement by other museum-goers, who occasionally listen in on the conversation. The disadvantages cited included the lost opportunity for informal social connections, as well as the serendipitous and tangential learning experiences that occurred in walking through the galleries between activities when the course was held in person. Although the students had described the course as being an appreciated deviation in learning experience from more traditional medical school courses, the TAs noted that in comparison to the in-person version of the course, the virtual course was a less immersive and mindful experience; on Zoom, the clock continues to tick by in the upper corner of the screen and students likely had distracting emails and other programs competing for their attention.

From a teaching perspective, the TAs viewed it as advantageous to be able to use digital features in zoom to enhance the viewing experience and to choose from a wider array of artwork. Additionally, they were able to use the chat function to field questions from the students during the lecture segments and to share their observations and supplemental resources with the rest of the class without interrupting a lecture or presentation. TAs noted that teaching the course over zoom posed a number of challenges in providing appropriately subtle guidance to students leading VTS sessions, in detecting reciprocal feedback from students based on the limited camera view, and in ascertaining student engagement. The TAs also reported that it was more difficult to connect personally with students in a mentorship role over Zoom.

Overall, despite the challenges of the new platform, the TAs unanimously appreciated improvement in students’ visual thinking and observational skills, in their own TA facilitation skills, and formation of camaraderie and friendships between participants.

### Faculty course directors

The course directors had similar observations to the TAs, with some different nuances. (Table 2) Perceived advantages of teaching TTE virtually included the ease of participation by all participants independent of geographic location, which logistically made it possible for faculty members who were frontline healthcare workers during the COVID-19 pandemic to participate despite demanding schedules, as well as for students who were no longer on campus. Using the world’s collection of artwork allowed for deliberate choices of diverse works that were most relevant to themes of each session; in addition to the collection at the MFA, artwork used for the VTS session included pieces from the Rijksmuseum, Amsterdam, The Netherlands; Whitney Museum of American Art, New York; Metropolitan Museum of Art, New York; and the Art Institute of Chicago.

Virtual viewing enabled remarkably detailed focus on artwork that one cannot get physically close enough to appreciate in real life. And, Zoom had a flattening effect on hierarchies inherent in medical education. Each individual, whether student, TA, or faculty, was represented as an equally sized square on the screen and projected at the same volume—an effect that disrupted typical patterns of potential deference and hesitation and actually worked well for developing team collaboration. While many of the students seemed more comfortable practicing VTS facilitation skills on Zoom than they had in person in years past, the course directors wondered if the skills acquired would translate to in-person visual interpretation and communication skills when the students were able to return to clinical sites.

The course directors also noted some drawbacks to the virtual format. Foremost was that the experience of the art galleries—the smells, echoes, ambience, and being together in that space —was unfortunately not recapitulated in a virtual environment, nor was the true experience of many pieces of art, whose scale, texture, and dimensions could not be translated to a flat screen. This provided an opportunity to draw parallels to the differences between in-person and telehealth medical encounters and examination. Efforts to actually incorporate patient examination were logistically more difficult, related both to the still relatively novel technology platform at that time as well as heightened medical issues for many of the past patient participants in the setting of the pandemic. With the one patient who joined the course virtually, students were much more hesitant to volunteer their observations in the virtual format than students of previous in-person course iterations; the subtle ability to read the patient’s body language and to make sure the patient could read theirs back may have been a significant barrier to engaging on this platform. Demonstrations of gait abnormalities also required more sophisticated camera management skills than our patients could manage.

Finally, and not unique to this course, the faculty had difficulty gauging how their presentations were being perceived by students and dynamically altering the session based on reciprocal interactions. We did not anticipate, and therefore, incorporate sufficient down-time to confirm ongoing student engagement and interaction.

Despite perceived limitations of the virtual class format, it was apparent to the course directors that the cognitive and emotional practices of VTS such as deep looking, active listening, and collaborative meaning-making were all taking place. When students led VTS sessions for the second time, they improved their delivery—a clear indication that students were achieving the course objectives. The course has always been conducted on a pass/fail basis, therefore direct comparison of grades was not possible. Engagement in the class is reliably high each year, and no students failed or dropped out of the course during the virtual session. Student sketchbook and journal entries are collected and reviewed by the faculty at the end of the course each year. The student entries in the study year subjectively demonstrated the same attention to detail, length of response, and level of self-reflection and insight as past years.

## Discussion

When TTE transitioned to a virtual platform in 2020, the course was successfully modified while maintaining the same objectives as the traditional class which had been offered previously for 17 years. In this paper, we discussed the evaluation of and insights on the course by students, TAs, and course faculty. From our post-course assessment we elucidated that there is much value in engaging in VTS in a virtual format, but that the experience for student and teacher alike differ from an in-person experience, and that there are aspects of teaching medical humanities virtually that could be improved upon.

Social and environmental interactions influence how humans think and problem-solve. With the online format, informal interactions were less common. Given that a goal of the course is to provide opportunities for mindful engagement, future emphasis should be placed on trying to foster relationships between all levels of participants. Potential solutions for this include keeping small groups of students together for multiple consecutive sessions, offering TA hours outside of class for informal student check-ins, creating informal and unscheduled post-class interactions between students and faculty to mimic the walk back to campus from the museum, and incorporating specific assessment activities to improve real-time monitoring of attainment of course goals. Virtual tours of art museums are increasingly available and may help give less acquainted students a perspective on art that is not otherwise captured in the virtual version of this course. The phenomenon of “Zoom fatigue” is all too familiar, and thus shortening session-length and the course in general, as well as adding stretch breaks and intentional reset moments may enhance the experience of and focus on the most critical components of the curriculum.

As a result of the pandemic, telehealth has become a staple of current and likely future medical care. Based on our first virtual iteration of this course, parallels can be drawn between the virtual art observation exercises and the paradigm of telemedicine. Virtual patient encounters require new methods of visual interpretation that can be harnessed to provide more compassionate, curious, and thorough care using remote platforms. Although the students were notably reticent in the single patient encounter in this course, familiarity tends to reduce apprehension. Desensitizing the students to this modality of evaluation by incorporating more such “encounters” would hopefully enhance their clinical skill set. We propose to add telemedicine simulations to future virtual humanities course sessions. Likewise, telemedicine providers may benefit from VTS teaching to improve the value and effectiveness of patient encounters.

This study is limited by its retrospective, qualitative nature, as well as the specific COVID-19 context that certainly was distracting to many participants. It is likely that virtual course offerings with more advanced planning may impact participation and satisfaction differentially; general knowledge regarding on-line teaching methods have significantly improved since the onset of the COVID-19 pandemic. Objective assessments of the students’ learning was not done and so the actual impact of the issues identified with remote learning remain unclear. Additionally, because student feedback was collected anonymously, we cannot assess any demographic differences in course experience, but it is possible that factors such as gender, race/ethnicity, or degree program affected the virtual course experience. And, as a single site study, the ability to generalize our findings may be limited.

Our medical humanities course was adapted for remote learning when in-person gatherings were suspended due to an unanticipated global pandemic in which medical curricula were mandatorily converted to a virtual format. Based on evaluation of the impact of our adaptations among various participants, we described the benefits and drawbacks to our teaching the course over Zoom, which may provide direction to similar efforts. A hybrid model may be able to optimize the balance between benefits of both teaching formats once it is safe to in person group learning.

## Supplementary Information

Below is the link to the electronic supplementary material.

Supplementary file1 (DOCX 15 kb)

Supplementary file2 (DOCX 15 kb)

Supplementary file3 (DOCX 17 kb)

## Data Availability

All data and materials comply with field standards and support the published claims.
