# Supplementary material for: Training the eye, virtually: adapting an art in medicine curriculum for on-line learning
Source: SN Soc Sci. 2022 Aug 11;2(8):158. doi: 10.1007/s43545-022-00442-4 (PMC9366826; doi:10.1007/s43545-022-00442-4)
Supplement: Supplementary file 1 — Supplementary file1 (DOCX 15 kb) [file 43545_2022_442_MOESM1_ESM.docx]

**Appendix A.** Annual post-course questionnaire

**Training the Eye: Improving the Art of Physical Diagnosis**

***Post-course Questionnaire***

Please respond to the following questions:

What does the phrase “making a diagnosis” mean to you?

Did this course meet your expectations? Please comment on how it did or did not.

What did you take away from the course?

Were there any particularly memorable or impactful readings, exercises, lectures or art pieces for you?

What did you enjoy most about the course?

What could be improved in the course?

Do you anticipate using Visual Thinking Strategies (VTS) in your clinical practice? If so, how?

What field(s) of medicine/dentistry are you considering at this point in your medical education?

After taking this course, do you expect your frequency of visiting an art Museum will increase, decrease, or remain the same (post-COVID)?

Would you recommend this course to peers? Why?

Any other comments:
