# Supplementary material for: Training the eye, virtually: adapting an art in medicine curriculum for on-line learning
Source: SN Soc Sci. 2022 Aug 11;2(8):158. doi: 10.1007/s43545-022-00442-4 (PMC9366826; doi:10.1007/s43545-022-00442-4)
Supplement: Supplementary file 2 — Supplementary file2 (DOCX 15 kb) [file 43545_2022_442_MOESM2_ESM.docx]

**Appendix B**. Additional on-line platform survey questions.

| Please rate how much you agree with the following statement.  I was able to meet the stated course objectives:   1. Make careful, objective observations a habit 2. Gain confidence in connecting form with function | | | |
| --- | --- | --- | --- |
| Strongly agree | Agree | Neutral | Disagree |
| Please rate how much you agree with the following statement.  The zoom platform worked well for Training the Eye. | | | |
| Strongly agree | Agree | Neutral | Disagree |
| Were there technical or other issues that hindered your experience? | | | |
| Were there certain aspects of the course that you found were advantageous using the online platform? | | | |
| How would you improve the course? | | | |
