# Supplementary material for: Training the eye, virtually: adapting an art in medicine curriculum for on-line learning
Source: SN Soc Sci. 2022 Aug 11;2(8):158. doi: 10.1007/s43545-022-00442-4 (PMC9366826; doi:10.1007/s43545-022-00442-4)
Supplement: Supplementary file 3 — Supplementary file3 (DOCX 17 kb) [file 43545_2022_442_MOESM3_ESM.docx]

**Appendix C**. Additional on-line platform interview questions for TAs and faculty.

**Training the Eye: Improving the Art of Physical Diagnosis**

***Teaching Assistant Interview Questions***

- How did you find the ease and effectiveness of the virtual format?
- What features of the remote class worked well and which did not?
- Did you experience any technical challenges?
- Did the remote interface impact ability to achieve the stated course objectives?
- What, if any, advantages did you perceive with the on-line course?
- Do you think you were able to connect to and help students in your role as TA?
- How well did you feel you were able to moderate the small group sessions remotely?
- How well do think students were able to practice using VTS on the remote platform?
- Do you have any suggestions for improving the virtual format of this course?
- What were the relative advantages of the remote platform compared to in-person class (having previously taken the course in person)?
- From a TA perspective, do you have a preference for continuing the virtual format of this course, in whole or in part, for future years?

***Faculty Interview Questions***

- How did you find the ease and effectiveness of the virtual format?
- What features of the remote class worked well and which did not?
- Did you experience any technical challenges?
- Did the remote interface impact ability to achieve the stated course objectives?
- What, if any, advantages did you perceive with the on-line course?
- Do you think you were able to connect to and teach students in your role as faculty?
- How well did you feel you were able to moderate the small group sessions remotely?
- How well do think students were able to practice using VTS on the remote platform?
- Do you have any suggestions for improving the virtual format of this course?
- What were the relative advantages of the remote platform compared to in-person class (having previously taken the course in person)?
- From a faculty perspective, do you have a preference for continuing the virtual format of this course, in whole or in part, for future years?
- Did you notice any ways in which student performance and/or achievement seemed to be impacted by conducting the course remotely?
